# Impact of Coronary Artery Disease on The Outcomes of Catheter Ablation in Patients with Atrial Fibrillation

**DOI:** 10.21470/1678-9741-2021-0537

**Published:** 2023

**Authors:** Fuqian Guo, Caiying Li, Chen Chen, Jiqiong Ni, Lan Yang, Yicheng Chen, Rong Fu, Yang Jiao, Yuanyuan Meng, Bulang Gao

**Affiliations:** 1 Department of Medical Imaging, The Second Hospital, Hebei Medical University, Shijiazhuang, Hebei, People’s Republic of China

**Keywords:** Atrial Fibrillation, Catheter Ablation, Recurrence, Coronary Artery Disease, Coronary Vessels, Treatment Outcome

## Abstract

**Introduction:**

The objective of this study is to investigate the possible impact of coronary artery disease (CAD) on clinical outcomes of catheter ablation in patients with atrial fibrillation (AF).

**Methods:**

Patients with AF who underwent coronary computed tomography and catheter ablation were enrolled. The presence of stenotic severity and plaque, characteristics of coronary arteries, clinical data, and adverse outcomes of catheter ablation were analysed.

**Results:**

A total of 243 patients were enrolled, 100 (41%) patients with CAD. The CHA_2_DS_2_-VASc (congestive heart failure, hypertension, age ≥ 75 years, diabetes mellitus, stroke or transient ischemic attack, vascular disease, age 65-74 years, and sex category) score of AF patients with CAD was significantly (*P*<0.001) higher than of those without CAD. Presence of stenotic artery and plaques increased significantly with increase of CHA_2_DS_2_-VASc score (*P*<0.05). There was no significant (*P*=0.342) difference in AF recurrence between patients with and without CAD (30% versus 24%). Age, AF type, duration of AF, heart failure, CHA_2_DS_2_-VASc score, left ventricular ejection fraction, and left atrial diameter were significantly (*P*<0.05) correlated with AF recurrence in univariant analysis. Multivariable analysis revealed that duration of AF (hazard ratio [HR] 1.769), heart failure (HR 1.821), and left atrial diameter (HR 1.487, *P*=0.022) remained significant independent predictors of AF recurrence. Patients with AF and concomitant CAD were significantly (*P*=0.030) associated with a worse outcome.

**Conclusion:**

CAD concomitant with AF may be associated with a worse clinical outcome even though CAD does not significantly affect the risk of AF recurrence after ablation therapy.

## INTRODUCTION

Atrial fibrillation (AF) is the commonest cardiac arrhythmia, with a high mortality, and is an independent risk parameter for stroke, necessitating early intervention to improve the prognosis^[1]^. Catheter ablation with pulmonary vein isolation (PVI) is currently considered as a safe and potential curative method, but the rates of AF recurrence remain as high as 30% to 60%^[2]^. Currently, several risk factors of AF recurrence have been identified, including persistent AF, left atrial size, hypertension, and obstructive sleep apnea^[3,4,5]^. Coronary artery disease (CAD) is not usually used to guide decision making regarding catheter ablation for AF. In clinical practice, AF and CAD frequently coexist, with the prevalence of CAD in AF population ranging from 36% to 82%, and coexistence of these two diseases leads to functional disability, a high mortality, and massive healthcare expenses^[6]^. Thus, it is necessary to investigate the effect of CAD on the AF prognosis outcome of catheter ablation in patients with CAD and AF. This study was consequently performed to investigate this effect by using computed tomography angiography (CTA) data and clinical outcomes of CAD in AF patients treated with catheter ablation.

## METHODS

### Study Design and Population

This study was approved by the Institutional Review Board of our hospital (No. 2018-R245). Written informed consent for participation was not required for this study in accordance with the national legislation and the institutional requirements because of its retrospective nature. Consecutive AF patients who experienced both CTA and AF ablation between October 2016 and September 2017 were retrospectively enrolled. The inclusion criteria were patients aged above 18 years, with symptomatic paroxysmal or persistent AF which was refractory to at least one antiarrhythmic drug, and with no history of catheter ablation or surgical ablation treatment. The exclusion criteria were valvular heart disease and coronary revascularization surgery.

CAD was defined as confirmed stenosis (≥ 50%) in at least one major coronary artery on CTA^[7]^. Electrocardiographic AF was defined as an irregular rhythm with fibrillatory waves but no defined P waves. Paroxysmal AF indicated AF which spontaneously terminated within seven days from onset, whereas persistent AF indicated continuous AF lasting more than seven days^[8]^. The stenotic severity and plaque characteristics of CAD and the incidence of postoperative major cardiovascular and cerebrovascular events were observed.

### CTA Data Acquisition

All patients underwent CTA scan using a 256-slice computed tomography scanner (Brilliance iCT, Philips Healthcare, Cleveland, Ohio, United States of America [USA]) during a breath-hold of approximately 4-7 seconds with retrospectively electrocardiography (ECG)-gated helical data acquisition. Patients with an average heart rate ≥ 65 beats per minute were given oral beta-blocker metoprolol (50-100 mg) 30-60 minutes before coronary CTA. Scan parameters were as follows: tube voltage, 120 kV; tube current, 280-350 mA; detector collimation, 128 × 0.625 mm; slice thickness, 0.67 mm; section interval, 0.33 mm; gantry rotation time, 330 ms; beam pitch, 0.18; pitch of 1; and a rotation time of 0.5 seconds. Automatic tube current modulation was used for all protocols as a default setting. Contrast material of iohexol (Omnipaque 350; 1.0 mL/kg) was injected into the ulnar vein at 4-5 mL/s.

### Image Analysis

The presence of atherosclerotic plaques and luminal narrowing were investigated using axial raw images, curved multiplanar reconstructions, and maximum intensity projection. Coronary artery segments were evaluated in accordance with the 15-segment American Heart Association classification^[9]^. Stenotic lesions were quantified in decrease of vascular lumen diameter and graded into four categories: none (0% stenosis), mild (1-49% stenosis), moderate (50-75% stenosis), and severe (≥ 75% stenosis)^[10]^. Significant coronary artery stenosis was defined as ≥ 50% stenosis. The prevalence of CAD in single-vessel, double-, or multivessel disease was evaluated; a left main lesion was defined as two-vessel coronary artery stenosis. To quantify the extent of CAD, segment involvement score was used, which indicated the sum of all segments with any plaque^[11]^. Subsequently, the atherosclerotic plaques were defined as structures > 1 mm^2^ within and/or adjacent to the coronary artery lumen, which could be clearly distinguished from the vessel lumen and the surrounding pericardial tissue. Plaques were defined as calcified with the density > 130 Hounsfield Unit (HU), non-calcified with the density < 130 HU compared with the contrast-enhanced vessel, and mixed ones with non-calcified and calcified elements within a single plaque^[12]^.

Image analysis was performed by two experienced radiologists (eight and six years of experience) who were blinded to all data. If disagreement existed between these two evaluators, a third observer (10 years of experience) was involved to decide.

### Echocardiographic Examination

All patients underwent transthoracic echocardiographic examination consisting of a standard two-dimensional echocardiogram (iE33, Philips Medical Systems, Bothell, Washington, USA), including M-mode and Doppler echocardiography to assess left ventricular systolic function.

### Ablation Procedure

Left atrial catheter ablation was performed using either radiofrequency ablation or cryoballoon ablation at the discretion of the physician. For radiofrequency ablation procedures, a 3.5-mm tip irrigated ablation catheter (Navistar Thermocool, Biosense-Webster, Diamond Bar, California, USA) was used and placed at the ostia of pulmonary vein (PV) to record PV potentials. For cryoablation procedures, a 28-mm cryoballoon catheter (Arctic Front, Advance TM, Medtronic Inc, Minneapolis, Minnesota, USA) was utilized to perform PVI. All patients received circumferential ipsilateral PVI with guidance of electroanatomic mapping (CARTO-3 system, Biosense-Webster, Diamond Bar, California, USA). The endpoint of the PVI was the bidirectional conduction block from the atrium to the PVs confirmed by Lasso catheter (Biosense-Webster, Diamond Bar, California, USA).

### Follow-up

Follow-up was performed at three, six, and 12 months to inquire about hospital admissions, cardiovascular and cerebrovascular events, and deaths. The endpoint at follow-up was recurrence of AF. AF recurrence was defined as atrial futter/atrial tachycardia > 30 seconds identified by surface ECGs or dynamic electrocardiogram beyond three-month blanking period after catheter ablation. Composite adverse events (all-cause death, heart failure, and stroke/transient ischemic attack [TIA]) were observed.

### Statistical Analysis

Continuous variables were presented as mean ± standard deviation for normal distribution, and median (interquartile range) for asymmetrically distributed data. Categorical variables are expressed as frequency and percentages. Differences between groups were evaluated by the Chi-square test for categorical variables and Student’s *t*-test or Mann-Whitney U test for continuous variables. Multivariable cox regression analyses with forwarding stepwise likelihood were performed to evaluate any factors associated with AF recurrence. Hazards and 95% confidence interval (CI) were calculated. Kaplan-Meier method was used to determine the event-free survival, and log-rank test was used to compare survival rate. All statistical analyses were performed using the IBM Corp. Released 2012, IBM SPSS Statistics for Windows, version 21.0, Armonk, NY: IBM Corp. All *P*-values were two-sided, and *P*-values < 0.05 were considered statistically significant.

## RESULTS

### Patients With and Without CAD

Two hundred forty-three patients were enrolled in the study (Table 1). CAD was present in 100 (41.2%) patients. Single-vessel disease was present in 60 (24.7%) patients, double-vessel disease in 28 (11.5%) patients, and a multivessel disease in 12 (4.9%) patients. The prevalence of hypertension, diabetes mellitus, male gender, age, and CHA_2_DS_2_-VASc (congestive heart failure, hypertension, age ≥ 75 years, diabetes mellitus, stroke or transient ischemic attack, vascular disease, age 65-74 years, and sex category) score were significantly (*P*<0.05) higher in CAD than in non-CAD group (*P*<0.05). A larger left atrium (*P*=0.019) and a small left ventricular ejection fraction (LVEF) (*P*=0.036) were present in patients with CAD than in those without CAD. No significant (*P*>0.05) differences existed in AF types and duration, body mass index, history of vascular disease, heart failure, stroke/TIA, hyperlipidemia, and smoking habit.

**Table 1:** Baseline characteristics of patients with and without coronary artery disease (CAD).

	AF-CAD (n=100)	AF-non-CAD (n=143)	*P*-value
Age (years)	63.46±8.64	58.7±10.06	< 0.001
Sex			
Male	71 (71%)	83 (58%)	0.039
Female	29 (29%)	60 (42%)	
AF type			0.058
Paroxysmal AF	80 (80%)	110 (77%)	
Persistent AF	20 (20%)	33 (23%)	
Duration of AF ≥ 1 year	68 (68%)	82 (57%)	0.093
Risk factors			
BMI (kg/m^2^)	26.33±3.08	26.54±3.36	0.618
Hypertension	72 (72%)	66 (46%)	< 0.001
Diabetes mellitus	26 (26%)	19 (13%)	0.012
Vascular disease	44 (44%)	46 (32%)	0.06
Hyperlipidemia	13 (13%)	16 (11%)	0.668
Current and previous smoking	26 (26%)	33 (23%)	0.601
Congestive heart failure	27 (27%)	25 (17%)	0.075
Previous stroke/TIA	23 (23%)	20 (19%)	0.07
CHA_2_DS_2_-VASc	2.80±1.51	1.99±1.43	< 0.001
Score 0	6 (6%)	13 (9%)	0.377
Score 1	18 (18%)	40 (28%)	0.073
Score ≥ 2	76 (76%)	90 (63%)	0.023
Echocardiography LVEF (%)	59.43±5.34	60.78±4.58	0.036
LA size (mm)			
Anteroposterior diameter	4.28±0.77	4.05±0.74	0.019
Superior-inferior diameter	5.62±0.70	5.65±0.81	0.762
Medial-lateral diameter	6.21±1.14	6.03±0.99	0.202
AF recurrence	31 (31%)	34 (24%)	0.343

AF=atrial fibrillation; BMI=body mass index; CHA_2_DS_2_-VASc=congestive heart failure, hypertension, age ≥ 75 years, diabetes mellitus, stroke or TIA, vascular disease, age 65-74 years, and sex category; LA=left atrial; LVEF=left ventricular ejection fraction; TIA=transient ischemic attack

The patients were divided into four groups according to age (Table 2). The prevalence of patients with CAD, stenotic extent, and myocardial ischemia had increased with increase of age, but it did not reach any significant difference (*P*>0.05).

**Table 2:** Prevalence of coronary artery disease (CAD) among different ages.

Age (years)	< 45	45-64	65-74	≥ 75	*P*-value
Number of patients	14	136	78	15	-
CAD	6 (43%)	53 (39%)	33 (42%)	8 (53%)	0.742
Single-vessel	3 (21%)	32 (24%)	18 (23%)	6 (40%)	0.592
Double-vessel	2 (14%)	15 (11%)	11 (14%)	1 (6%)	0.829
Multivessel	1 (7%)	6 (4%)	4 (5%)	1 (6%)	0.662
Stenotic extent					
Moderate (50-75%)	3 (21%)	30 (22%)	21 (30%)	4 (27%)	0.885
Severe (≥ 75%)	3 (21%)	23 (17%)	12 (15%)	4 (27%)	0.797
MI	1 (7%)	22 (16%)	10 (13%)	4 (27%)	0.470

MI=myocardial ischemia

**Fig. 1:**
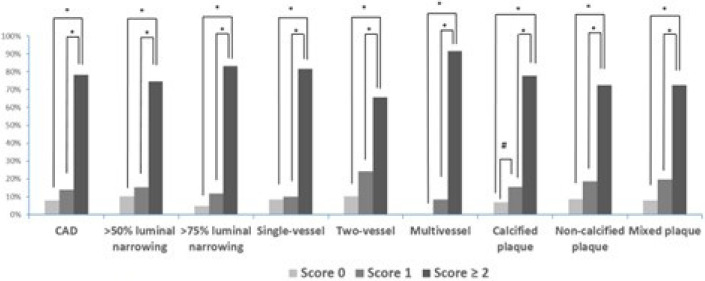
Prevalence of coronary artery disease (CAD) and total number of segments containing non-calcified, calcified, or mixed plaques in atrial fibrillation patients among three groups classified by CHA_2_DS_2_-VASc (CHA_2_DS_2_-VASc=congestive heart failure, hypertension, age ≥ 75 years, diabetes mellitus, stroke or TIA, vascular disease, age 65-74 years, and sex category) scores 0, 1, and ≥ 2. *P<0.001; #P=0.037.

After 243 patients were divided into three groups according to the CHA_2_DS_2_-VASc score (0, n = 33 [13%]; 1, n = 48 [20%]; ≥ 2, n = 162 [67%]), the prevalence of CAD and total number of segments containing atherosclerotic plaques were significantly increased with greater CHA_2_DS_2_-VASc scores (*P*<0.05) (Figure 1).

In our study, patients with suspected CAD underwent a stress or exercise test, and myocardial ischemia was detected in 37 (37%) patients. Among 100 patients with coronary artery stenosis > 50% and the remaining 143 patients without stenosis or with stenosis < 50%, no patient had serious adverse cardiovascular events or acute coronary syndrome before and during radiofrequency ablation for AF. After radiofrequency ablation, 28 of 37 patients with myocardial ischemia were treated by optimal medical therapies, and only nine patients received re-vascularization of coronary arteries with percutaneous coronary intervention (PCI).

### Clinical Follow-Up

Catheter ablation procedure was successful in all patients (100%). During a median follow-up of 27 months (Q1-Q3: 18-31 months), AF recurrence rate was 27% (64/243) in all patients, 30% in patients with CAD, and 24% in patients without CAD, with no significant (*P*=0.342) difference between patients with and without CAD. The stenotic lesions and plaque characteristics of coronary artery stenosis were analyzed in different groups based on CTA (Table 3). There was no significant (*P*>0.05) difference in the proportion of arterial stenosis (single, double, or multiple vessels), the segment involvement score, or plaque characteristics of CAD between patients with and without AF recurrence. Patients with AF recurrence had significantly (*P*=0.025) more diseases in the left main stem. Patients with coronary artery lesions in the proximal right coronary artery (RCA), left anterior descending artery (LAD), and left circumfex artery (LCX) showed a higher trend towards AF recurrence but without reaching a statistical significance (*P*=0.057).

**Table 3:** Stenotic severity and plaque characteristics of coronary artery diseases in patients with and without atrial fibrillation (AF) recurrence.

Angiographic parameter	AF recurrence (n=65)	Non-AF recurrence (n=178)	*P*-value
Single-vessel	18 (28%)	41 (23%)	0.453
Double-vessel	9 (14%)	20 (11%)	0.579
Multivessel	4 (6%)	8 (4%)	0.846
SIS	0.95±1.37	0.84±1.43	0.570
Stenotic extent			
None (0%)	28 (43%)	62 (35%)	0.239
Mild (1-49%)	14 (22%)	37 (21%)	0.899
Moderate (50-75%)	17 (26%)	43 (24%)	0.749
Severe (≥ 75%)	6 (9%)	36 (20%)	0.265
Distribution			
Left main artery	4 (6%)	2 (1%)	0.025
LAD	26 (40%)	56 (31%)	0.213
Proximal	23 (35%)	46 (26%)	0.144
Mid	11 (17%)	27 (15%)	0.739
Distal	2 (3%)	9 (5%)	0.511
LCX	9 (14%)	19 (11%)	0.493
Proximal	9 (14%)	16 (9%)	0.27
Distal	0	3 (2%)	0.566
RCA	12 (18%)	29 (16%)	0.689
Proximal	8 (12%)	23 (13%)	0.899
Mid	3 (5%)	12 (7%)	0.758
Distal	2 (3%)	11 (6%)	0.529
Proximal lesions*	40 (62%)	85 (48%)	0.057
Plaque characteristics			
Calcified	33 (5%)	83 (46%)	0.567
Non-calcified	22 (34%)	58 (33%)	0.853
Mixed	16 (25%)	35 (20%)	0.401

* Proximal lesions include proximal RCA, left main artery, proximal LAD, and proximal LCX

LAD=left anterior descending artery; LCX=left circumfex artery; RCA=right coronary artery; SIS=segment involvement score

**Fig. 2:**
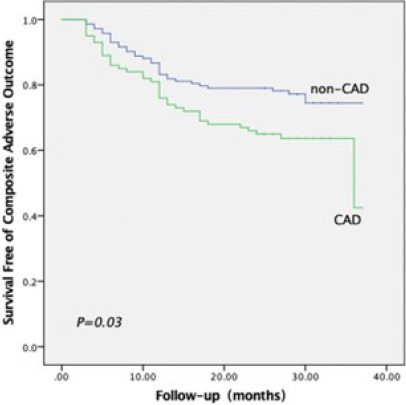
Kaplan–Meier survival curves for the composite adverse outcome during a median follow-up of 27 months. The event-free survival rate was significantly (P=0.03) worse in patients with atrial fibrillation and coronary artery disease (CAD).

Univariate cox analysis indicated that age, AF type, duration of AF, heart failure, CAD, CHA_2_DS_2_-VASc score, LVEF, and left atrial anteroposterior diameter were significantly (*P*<0.05) correlated with AF recurrence. Multivariable analysis revealed that duration of AF (hazard ratio [HR] 1.769, 95% CI: 11.027, 3.048, *P*=0.040), heart failure (HR 1.821, 95% CI: 1.067, 3.107, *P*=0.028), and left atrial diameter (HR 1.487, 95% CI 1.059, 2.088, *P*=0.022) remained independent predictors of AF recurrence. However, no significant (*P*>0.05) correlation was found in CAD and AF recurrence (Table 4). Composite adverse events included 74 events in patients with CAD (one cardiac death, three strokes/TIAs, three heart failures, 31 recurrences of AF) and without CAD (two strokes/TIAs, 34 recurrences of AF). In Kaplan–Meier curve analysis for comparison of event-free survival from the composite endpoint events, patients with AF and concomitant CAD were associated with a significantly worse outcome (*P*=0.030; Figure 2).

**Table 4:** Univariable and multivariable regression analyses of recurrence of atrial fibrillation (AF).

Variables	Non-AF recurrence	AF recurrence	Univariant analysis			Multivariant analysis		
			HR	95% CI	*P*-value	HR	95% CI	*P*-value
Age (years)	60.12±9.69	62.18±9.89	1.019	(0.992, 1.047)	0.016	NA	NA	NA
Sex (male)	114	40	1.082	(0.657, 1.784)	0.756	NA	NA	NA
Persistent AF	32	20	1.712	(1.011, 2.901)	0.045	NA	NA	NA
Duration of AF	103	47	1.732	(1.006, 2.982)	0.048	1.769	(1.027, 3.048)	0.040
Hypertension	98	40	1.278	(0.775, 2.107)	0.336	NA	NA	NA
Diabetes mellitus	35	10	0.754	(0.384, 1.476)	0.411	NA	NA	NA
Previous stroke/TIA	29	14	1.302	(0.721, 2.353)	0.381	NA	NA	NA
Congestive heart failure	31	21	2.061	(1.224, 3.468)	0.006	1.821	(1.067, 3.107)	0.028
Vascular disease	59	31	1.589	(0.977, 2.586)	0.062	NA	NA	NA
Hyperlipidemia	18	11	1.596	(0.835, 3.054)	0.158	NA	NA	NA
CHA2DS2-VASc	2.2±1.4	2.7±1.5	1.228	(1.042, 1.448	0.014	NA	NA	NA
Current and previous smoking	47	12	0.672	(0.359, 1.257)	0.214	NA	NA	NA
LVEF	60.6±4.3	59.1±6.2	0.949	(0.910, 0.989)	0.013	NA	NA	NA
LA anteroposterior diameter	4.04±0.75	4.3±0.73	1.595	(1.147, 2.217)	0.005	1.487	(1.059, 2.088)	0.022
CAD	69	31	1.391	(0.855, 2.263)	0.184	NA	NA	NA

CAD=coronary artery disease; CHA_2_DS_2_-VASc=congestive heart failure, hypertension, age ≥ 75 years, diabetes mellitus, stroke or TIA, vascular disease, age 65-74 years, and sex category; CI=confidence interval; HR=hazard ratio; LA=left atrial; LVEF=left ventricular ejection fraction; TIA=transient ischemic attack

## DISCUSSION

In this study, the effect of CAD on the AF prognosis outcome of catheter ablation in patients with CAD and AF was investigated, and it was found that presence of CAD did not significantly affect AF recurrence after catheter ablation even though the CHA_2_DS_2_-VASc score increase was correlated with the prevalence and severity of stenosis of the coronary artery.

On the basis of CTA, coronary narrowing in patients with AF who undergo radiofrequency catheter ablation could be detected at the time of image acquisition of left atrial and pulmonary veins. As it is well-known, the prevalence of CAD increases with age. Moreover, coronary artery lesions also significantly increase in accordance with the CHA_2_DS_2_-VASc score. High CHA_2_DS_2_-VASc scores have been shown to be a predictor of cardio- and cerebrovascular events in patients with coronary heart disease^[13]^, and primary prevention measures through diet, exercise, optimal drug treatment including statins, and revascularization in symptomatic CAD patients may reduce the risk of future cardiovascular events. CAD frequently coexists with AF and can adversely affect the prognosis of ablation for AF. The high prevalence of CAD in patients with AF clearly shows that atherothrombosis plays a key role in the development of AF^[14,15]^. So far, several preclinical studies^[16,17]^ have examined the underlying relationship between coronary obstruction and risk of AF, which suggested that atrial ischemia could trigger myocardial damage, subsequent fibrosis, and scarring of atrial wall, resulting in reduced or even blocked conduction and subsequent facilitation of atrial arrhythmias. Given the anatomical characteristics of atrial branches, the main arterial supply comes either from the sinoatrial node or atrioventricular nodal arteries, which can vary between the left circumfex branch and the RCA^[18]^. AF complicated with CAD may be related to the location of diseased vessels and vascular lesions. Alexander et al.^[19]^ found that significant coronary artery lesions in the proximal RCA, LCX, LAD, and posterior descending artery were significantly associated with new-onset AF within one year. Vinter et al.^[14]^ reported that a high coronary artery calcium score was associated with a high risk of subsequent AF development. Thus, knowing the state of coronary atherosclerosis helps to prevent adverse clinical outcomes.

Currently, the impact of atrial ischemia caused by coronary artery lesion on the eficacy of AF treatment is still controversial. Kornej et al. demonstrated that in 152 patients (11.6%) with CAD, among 1,310 consecutive patients, no significant (*P*>0.05) correlation was found in the location and severity of CAD with the rhythm outcomes after CA, whereas the traditional risks of AF type and left atrial size were significant predictors^[20,21]^. Similar findings were observed in our cohort with CAD and AF. However, Daigo et al.^[22]^ found that AF recurrence was significantly higher in patients with CAD (56%) than in those without CAD (39%), but significantly lower in patients with PCI (38%) than in those without PCI (72%). CAD in the RCA, especially in the proximal segment, was more frequently detected in patients with PCI^[22]^. However, no significant (*P*>0.05) differences were detected in the distribution and plaque characteristics of major coronary arteries stenosis between patients with and without recurrence in our study. Moreover, the AF recurrence rate was 30% in patients with CAD but 24% in patients without CAD, which was quite lower than in those in the report by Daigo et al.^[22]^. This difference may be caused by the varied extents of coronary artery stenosis and fibrosis of patients in our study as compared with theirs. Our multivariable model showed that the left atrial diameter and duration of AF were significantly associated with AF recurrence, which was consistent with a previous study^[23]^. This may suggest that long-term exposure to elevated pressures potentially leads to autonomic structural and electrical changes in the atrium. Heart failure was also found to affect AF recurrence in our study, which was probably caused by myocardial fibrosis or atrial remodeling^[24]^.

It has been reported that 5.7% of patients who had undergone catheter ablation for AF sufered cardiovascular comorbidities during the subsequent year^[25]^. Postoperative AF was associated with increased stroke and death after discharge^[26]^. Other authors found that CAD with AF was significantly associated with an increased risk of mortality and adverse cardiovascular events including thromboembolism and heart failure^[27]^. In our study, higher incidences of stroke and heart failure were present in CAD patients. Therefore, AF patients with CAD are at a higher risk of developing cardiovascular and cerebrovascular events. If the involvement of CAD is recognized at an early stage, statin or antiplatelet drugs should be given in addition to anticoagulant therapy so as to prevent adverse cardiovascular events.

### Limitations

Our study had a few limitations, including its single-center and retrospective nature, Chinese patients enrolled only, and inclusion of concomitant diseases such as diabetes mellitus and hypertension, which may all contribute to publication bias. Moreover, it included patients who underwent both radiofrequency ablation and cryoablation for AF, which may, to some extent, influence the AF ablation outcomes. Future studies will have to solve these issues for better outcomes.

## CONCLUSION

In conclusion, CAD concomitant with AF may be associated with a worse clinical outcome even though CAD does not significantly affect the risk of AF recurrence after ablation therapy.
